# Circular RNAs as a Diagnostic and Therapeutic Target in Cardiovascular Diseases

**DOI:** 10.3390/ijms24032125

**Published:** 2023-01-21

**Authors:** Victor Hugo Antonio Joaquim, Noemy Pinto Pereira, Tiago Fernandes, Edilamar Menezes Oliveira

**Affiliations:** Laboratory of Biochemistry and Molecular Biology Applied to the Exercise, School of Physical Education and Sport, University of Sao Paulo, Sao Paulo 05508-030, Brazil

**Keywords:** circRNAs, cardiovascular diseases, physical exercise

## Abstract

Circular RNAs (circRNAs) are a family of noncoding RNAs (ncRNAs) that are endogenous and widely distributed in different species, performing several functions, mainly their association with microRNAs (miRNAs) and RNA-binding proteins. CVDs remain the leading cause of death worldwide; therefore, the development of new therapies and strategies, such as gene therapies or nonpharmacological therapies, with low cost, such as physical exercise, to alleviate these diseases is of extreme importance for society. With increasing evidence of ncRNA participating in the progression of CVDs, several studies have reported these RNAs as promising targets for diagnosis and treatment. There are several studies of CVDs and the role of miRNAs and lncRNAs; however, little is known about the new class of RNAs, called circRNAs, and CVDs. In this mini review, we focus on the mechanisms of circRNAs and CVDs.

## 1. Biogenesis, Characteristics, and Function

The first circRNA molecule that is covalently closed was discovered by Sanger et al., (1976) [1] in plant viroids; since then, some studies associated with circRNAs have been published. However, only in 2010 with advances in RNA sequencing technology did several studies emerge linking circRNAs to diseases such as cardiovascular diseases (CVDs) and cancer [2,3,4,5,6,7,8,9].

**Figure 1:**
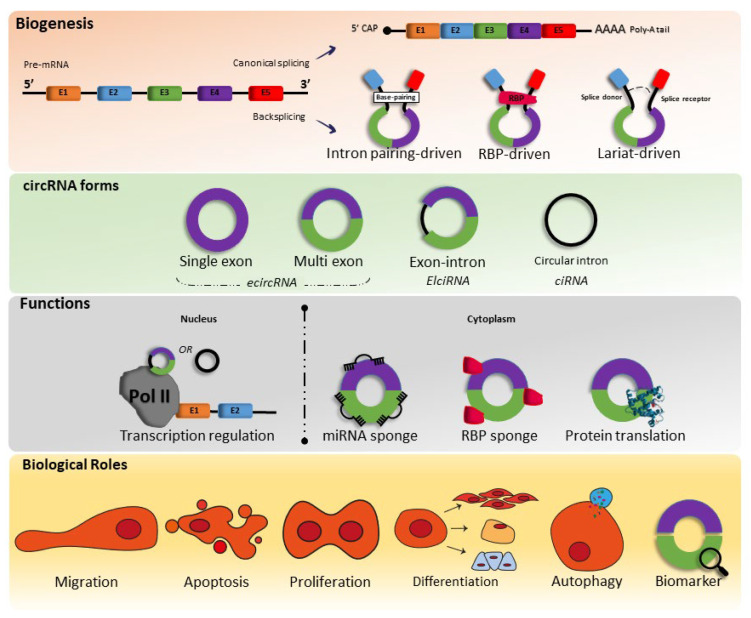
Biogenesis, forms, functions, and biological roles of circRNAs. miRNA—microRNAs; RBP—RNA-binding proteins.

CircRNAs are formed through pre-mRNA backsplicing; during backsplicing, a downstream 5′ splice site is linked to an upstream 3′ splice site in a reverse orientation, thus resulting in a circular RNA molecule with a 3′,5′ phosphodiester bond at the backsplicing junction site [10]. This process can occur both at the co- and posttranscriptional levels [10,11,12]. However, the circular RNA molecule can be formed in several ways, and until recent studies, are divided into five categories: exonic circular RNA (EcircRNA), where most are located in the cytoplasm, intronic circular RNA (ciRNA), exon–intron circular RNA (EIcircRNA), where the latter two are mostly located nucleus fusion circRNAs (f-circRNAs), and read-through circRNAs (rt-circRNAs) [10,11] (Figure 1).

Therefore, as circRNAs are generated from pre-mRNA backsplicing, as mentioned above, the literature shows that there are three types of models of biogenesis, including intron-pairing-triggered circularization, RNA binding protein (RBP)-driven circularization, and lariat-triggered circularization.

Circularization driven by intron pairing is considered the primary model for the formation of circRNA, and intronic complementary sequences (ICSs), such as Alu elements, which are known as transposable elements that flank backsplicing exons, are joined by base pairing, which triggers the formation of circRNA and exon-intron-containing circular RNA (EIcircRNA). In RNA-binding protein (RBP)-driven circularization, RBPs recognize unique motifs in the pre-mRNA to initiate circRNA formation. The docking of RBPs into ICSs promotes or inhibits circRNA formation. In lariat-driven circularization, intronic lariats that escape debranching and degradation can be processed into circular intronic RNA (ciRNA) during splicing [8,9,12].

CircRNAs have multiple functions, including [1] regulating transcription mediated by polymerase II (Pol II), [2] acting as miRNA sponges to block inhibitory effects on mRNAs, [3] sponging proteins to suppress their activity, or serving as scaffolds to bring in proteins to promote protein activity, [4] encode peptides, and proteins, and [5] serve as biomarker (Figure 1).

The role of circRNA as miRNA sponge is currently one of the most studied. When the miRNA performs partial base pairing with the 3′ UTR of its complementary mRNA, it promotes translational repression. Some research has reported the existence of miRNA binding sites on circRNA. It has been shown that circRNA can be a competing endogenous RNA (ceRNA) that binds to miRNA, thus weakening the inhibitory effect of miRNA on mRNA but promoting the expression of target genes [13]. This process is the “effect sponge” from circRNA. However, it will only work when the abundance of circRNA that has a sponge effect matches the abundance of miRNA. A single circRNA can bind many miRNAs at one or more sites and can have opposite effects on different diseases.

## 2. circRNA Expression in Cardiovascular Diseases

The study of circRNAs has gained strength with the advancement of transcriptomics analysis technologies and has become crucial to understand the molecular mechanisms of the pathophysiology of several health conditions. CVDs are the leading cause of death worldwide [14], making the cardiovascular system one of the main focuses of attention in circRNA studies, showing an emerging body of evidence about the ubiquitous role of this class of ncRNA at the posttranscriptional level [15].

CircRNAs can regulate gene expression in CVDs usually through a circRNA-miRNA-mRNA axis [16], acting as ceRNAs (“miRNA sponges”) or as protein antagonists, modulating several biological processes, such as cell proliferation, migration, differentiation, and apoptosis [17].

Using RNA sequencing and specific bioinformatics tools, Tan et al., [2] found 15,318 and 3017 circRNAs expressed in 12 human and 25 mouse hearts, respectively, across a 28-day differentiation time course. In human hearts, 82.32% of identified circRNAs were exonic and generated from their respective linear cognate gene. Among these, the most abundant cardiac-expressed circRNA was generated from the SLC8A1 gene (or NCX1 gene, from Na^+^/Ca^++^ exchanger), found in all 12 heart samples and formed by a single exon. The following top 10 highest circRNAs expression ranking found in all human heart samples also contains circHIPK3-2, circEXOC6B-14, circALPK2-2, and circMB-2. A comprehensive analysis of RNA-seq collected from two databases found 15,278 circRNAs in five human adult heart samples and 797 circRNAs in four mouse heart samples [18]. They also found 1608 circRNAs in three human fetus samples, reinforcing the evidence that the abundance of circRNAs seems to vary throughout biological development [19]. In Fischer 344 rats, Gong et al., [20] found that a significant number of circRNAs were differentially expressed depending on the organ (across 11 organs), sex (male and female), and age (juvenile, adolescence, adult, and aging). Additionally, when investigating the translational landscape of 80 human hearts, van Heesch et al., [21] found 8878 circRNAs in 3181 genes. The most interesting finding of this study was that 40 of these circRNAs had the potential to translate into proteins due to their ribosome association, a function considered rare but previously reported [22]. 

## 3. circRNAs in Arterial Hypertension

Arterial hypertension (AH) is characterized by a chronic and abnormal rise in blood pressure, with a multifactorial etiology involving genetic, environmental, and social determinants, and is the main contributor to the global burden of CVDs [23].

Liu et al., [24] found 485 differentially expressed circRNAs in aortic vascular tissue of spontaneously hypertensive rats (SHR) when compared to Wistar Kyoto (WKT) rats. Three circRNA-miRNA-mRNA axes were predicted and later confirmed in SHR aortas, regulating the NOTCH1, FOXO3, and STAT3 genes, all of which are involved in vascular diseases. The researchers highlighted that some of these differentially expressed circRNAs found in rats were highly similar to homologous sequences in human circRNAs. In another study, researchers used a model of angiotensin-II-induced vascular smooth muscle cell senescence and vascular samples from human hypertensive patients to demonstrate that the overexpression of circACTA2 decreased CDK4 mRNA stability and protein expression, leading to cell senescence [25]. Endothelial dysfunction, crucial for the development of arterial hypertension, is closely associated with several ncRNAs, especially lncRNAs, circRNAs, and miRNAs [26]. CircACTA2 may also be involved in vascular function and remodeling by regulating smooth muscle alpha actin (αSMA) expression [27,28].

To date, the human circRNAs_0037911, _0126991, and _0005870 seem to be the best candidates as hypertension biomarkers. CircRNA_0037911 and _0126991 were found to be highly regulated in the blood of hypertensive patients, while circRNA_0005870 was downregulated. Taken together, these circRNAs seem to be involved in vascular endothelial dysfunction and, consequently, the development of arterial hypertension (Table 1).

## 4. circRNAs in Myocardial Infarction

Myocardial infarction (MI) is a highly prevalent CVD with substantial global morbidity and mortality rates [37], making MI one of the main focuses of circRNA-related studies, with a large number of described circRNAs [38] (Table 2). Ischemia/reperfusion (I/R) injury is one methodology to study the mechanisms that lead to tissue injury in this disease, and we have had a huge increase in the understanding of how ncRNAs are involved in several underlying pathological mechanisms in IR injury, acting as biomarkers or as therapeutic strategies [39,40,41], especially those related to myocardial inflammation, apoptosis, angiogenesis, fibrosis, and oxidative damage.

With a known key role in fibrotic and tumoral diseases [42], circHIPK3, which is highly expressed in several human tissues, seems to be involved in even more pathophysiological processes. Si et al., [43] showed the cardiac regeneration effect of circHIPK3 by modulating cardiomyogenesis and angiogenesis after MI. When overexpressed, circHIPK3 increased the proliferation, migration, and tube formation of coronary vessel endothelial cells, attenuated cardiac dysfunction, and decreased the MI-related fibrotic area. Researchers conclude that circHIPK3 may be an ideal candidate as a therapeutic target to improve prognosis after MI. Another study also found that circHIPK3 regulates post-MI cardiac angiogenesis, but in cardiomyocyte-derived exosomes [44].

Myocardial-infarction-associated circular RNA (MICRA), from the ZNF604 locus, remains the main circRNA biomarker associated with MI. Salgado-Somoza et al., [45] used the blood levels of MICRA to stratify patients into groups depending on how preserved their left ventricular ejection fraction (LVEF) was four months after MI. MICRA was able to improve the risk classification and was proposed as a biomarker in the prognostication strategy. In another study, Vausort et al., [46] enrolled infarcted patients from two independent cohorts and found that MI patients had lower MICRA expression levels than healthy subjects. They also indicated MICRA as a strong predictor of left ventricle dysfunction.

**Table 2:** List of circRNAs associated with myocardial infarction and I/R injury.

Id	Host Gene	Species	Source	Expression	Action	Ref.
Cdr1as/CiRS-7	CDR1	Mouse	Cardiomyocytes	Up	Promotes CM apoptosis by sponging miR-7a.	[47]
		Pig	Myocardium	Up	It is positively correlated with better left and right ventricle function and infarcted size decrease.	[48]
circFndc3b	FNDC3B	Mouse Human	Cardiomyocytes	Down	Limits ischemic injury via FUS/VEGF signaling.	[49]
circNfix	Nfix	Mouse	Cardiomyocytes	Up	Promotes cardiac regenerative repair after MI by suppressing Ybx1 degradation and increasing miR-214 activity.	[50]
circ-Ttc3	TTC3	Rat	Cardiomyocytes	Up	Promotes cardioprotection via miR-15b/Arl2 axis.	[51]
MICRA	ZNF609	Human	Blood	Down	Biomarker.	[45]
circFASTKD1	FASTKD1	Human	HUVECs, HCMECs	Up	Its downregulation improved cardiac function after MI by enhancing angiogenesis via miR-106a/LATS1/2/YAP pathway.	[52]
circHelz	Helz	Mouse	Myocardium NMVCs	Up	Promotes pyroptosis resulting in myocardial injury via miR-133a-3p/NLRP3 axis.	[53]
circJARID2	Jarid2	Mouse	Cardiac tissues	Up	Apoptotic and inflammatory damage in CM promoted by miR-9-5p/BNIP3 axis.	[54]
circ-100338	SNX27	Human	HCAEC	Down	Induces angiogenesis in I/R injury by sponging miRNA-200a-3p.	[55]
circDLGAP4	HECTD1	Mouse	HUVECs	Down	Plays a role in apoptosis and cell migration via miR-143/HECTD1 axis.	[56]
circ-SWT1	SWT1	Human	HC AC16 cells	Down	Reduces apoptosis, oxidative stress, and endoplasmic reticulum stress via miR-192-5p/SOD2 axis.	[57]
circROBO2	Robo2	Mouse	Myocardial tissues	Up	circROBO2 knockdown reduces apoptosis by sponging miR-1184 and enhancing TRADD expression levels.	[58]
circRNA-101237	CDK8	Mouse	Cardiomyocytes	Up	Regulates CM apoptosis via let-7a-5p/IGF2BP3 axis.	[59]
circPAN3	PAN3	Rat	Cardiac tissues	Up	Promotes cardiac fibrosis after MI via miR-221/FoxO3/ATG7 axis.	[60]
circNCX1	NCX1	Mouse	Cardiomyocytes	Up	Promotes CM apoptosis via miR-133a-3p/CDIP1 regulatory pathway.	[61]
circRNA1615	Copb1	Mouse	Myocardial tissue	Down	Modulates autophagy by the miRNA152/3p/LRP6 molecular axis, reducing ferroptosis in MI mouse hearts.	[62]
circ_0023461	ARAP1	Human	HC AC16 cells	Up	Reduces hypoxia-induced dysfunction in cardiomyocytes via miR-370-3p/PDE4D axis.	[63]
circHipk3	Hipk3	Mouse	Cardiomyocyte	Up	Triggers CM proliferation and angiogenesis by miR-133a/Notch1 signaling path.	[43]
		Mouse	Cardiomyocyte	Up	Induces tube formation, cell proliferation, and migration via miR-29a/VEGFA axis, stimulating cardiac angiogenesis after MI.	[44]
		Human	HCM	Up	Induces CM apoptosis after I/R injury by sponging miRNA-124-3p.	[64]
circTLK1	TLK1	Mouse	Cardiomyocyte	Up	Promotes CM apoptosis via miR-214/RIPK1-mediated TNF signaling pathway.	[65]
MFACR	Smyd4	Mouse	Cardiomyocyte	Up	Induces CM apoptosis via miR-652-3p/MTP18 axis.	[66]
		Human	Plasma, HC AC16 cells	Up	Promotes CM apoptosis by downregulating miR-125b through methylation.	[67]
circUbe3a	Ube3a	Mouse	Cardiac tissue	Up	Exacerbates MI-induced myocardial fibrosis via miR-138-5p/RhoC axis.	[68]
circ_0060745	Cse1l	Mouse	Cardiomyocyte	Up	Increases infarct size and impaired cardiac function after MI.	[69]
ACAP2	ACAP2	Human	Plasma, HC AC16 cells	Up	Induces CM apoptosis by promoting maturation of miR-532.	[70]
circCDYL	CDYL	Mouse	Cardiomyocyte	Down	Promotes CM proliferation through miR-4793-5p/APP pathway.	[71]
circTRRAP	TRRAP	Human	HC AC16 cells	Up	Increases inflammatory, apoptotic, and oxidative damage in CM via miR-370-3p/PAWR axis.	[72]
circMACF1	Macf1	Mouse	Cardiomyocyte	Down	Attenuates CM apoptosis via miR-500b-5p/EMP1 axis.	[73]
circACR	-	Mouse	Cardiomyocyte	Down	Promotes cardioprotection, decreasing myocardial infarction size, autophagy, and cell death via Pink1/FAM65B axis.	[74]
circMAT2B	MAT2B	Rat	H9c2 cells	Up	circMAT2B knockdown promotes anti-inflammatory and antiapoptotic role via miR-133/PI3K/AKT and Raf/MEK/ERK pathways.	[75]
circNFIB	Nfib	Mouse	Cardiac fibroblast	Down	Reduces cardiac fibrosis after MI by sponging miR-433.	[76]

MI—myocardial infarction; CM—cardiomyocyte; I/R—ischemia/reperfusion; HUVECs—human umbilical vein endothelial cells; HCMECs—human cardiac microvascular endothelial cells; HCM—human-derived cardiomyocytes; NMVCs—neonatal mouse ventricular cardiomyocytes; HCAEC—human coronary endothelial cells; HC AC16 cells—human cardiomyocytes AC16 cells.

## 5. circRNAs in Coronary Artery Disease

Additionally, known as coronary heart disease or even ischemic heart disease, coronary artery disease (CAD) is characterized by atherosclerotic occlusions of the coronary arteries, possibly leading to myocardial infarction and sudden death, and it is the primary cause of death worldwide [77,78]. Conditions such as chronic inflammation and endothelial injuries are involved in the complex etiology of CAD, and some circRNAs have been linked with the development and progression of this disease [79] (Table 3). 

In a mouse model study, Gong et al., [80] reported the role of circEsyt2 in vascular remodeling by enhancing cell proliferation and migration while inhibited apoptosis and differentiation in vascular smooth muscle cells (VSMC). The experimental findings were in accordance with the higher circEsyt2 expression found in severe-CAD patients when compared to mild-CAD patients, suggesting a proatherosclerotic role of this circRNA. Inversely, circANRIL was found to induce apoptosis and inhibit proliferation, conferring atheroprotection [81]. The most interesting finding here is that both linear and circular ANRIL were differentially expressed in CAD patients vs. controls, but linear ANRIL showed an atheros progressive function.

Several circRNAs have been proposed as CAD biomarkers thus far. In CAD vs. non-CAD subjects, the combined expression of circRNAs hsa_circ_0001879 and hsa_circ_0004104 showed equal diagnostic value compared to Holter monitoring, treadmill exercise tests, and coronary computed tomography angiography [82]. hsa_circ_0004104 may also be involved in CAD pathogenesis. hsa_circ_0124644 is also a promising CAD biomarker. In a cohort study, hsa_circ_0124644 was upregulated (2.2-fold change) in the CAD group (n = 137) vs. control group (n = 115). In the CAD vs. control scenario, hsa_circ_0124644 expression had a sensitivity and specificity of 0.86 and 0.62, respectively. The subjects from the CAD group were then classified into two different groups according to disease severity, and hsa_circ_0124644 retained its diagnostic value [83].

**Table 3:** List of circRNAs associated with CADs.

Id	Host Gene	Species	Source	Expression	Action	Ref.
hsa_circ_0001879	NIPSNAP3A	Human	Peripheral blood	Up	Biomarker.	[82]
hsa_circ_0004104	SPARC	Human	Peripheral blood	Up	Biomarker. Its upregulation might contribute to the pathogenesis of atherosclerosis.	
circ-YOD1	YOD1	Human	Blood/HASMCs	Up	Biomarker.	[84]
hsa_circ_0124644	ROBO2	Human	Peripheral blood	Up	Biomarker.	[83]
circEsyt2	Esyt2	Mouse	Aortae tissue	Up	Enhances cell migration and proliferation and inhibits apoptosis and differentiation in VSMC.	[80]
circANRIL	ANRIL	Human	Peripheral blood	Down	Promotes atheroprotection by increasing apoptosis and inhibiting macrophages proliferation.	[81]
hsa_circ_0001445	SMARCA5	Human	Plasma	Down	Biomarker.	[85]
hsa_circ_0005540	MCTP1	Human	Plasma	Up	Biomarker.	[86]

HASMCs—human aortic smooth muscle cells; VSMC—vascular smooth muscle cells.

## 6. circRNAs in Abdominal Aortic Aneurysm

Although there is a lack of consensus, abdominal aortic aneurysm (AAA) could be defined as a greater than 1.5-fold increase in a localized region of the abdominal aorta diameter, with a slow expansion until its rupture [87]. It is estimated that AAA causes 150,000–200,000 deaths per year worldwide, with higher prevalence in the male, smoker, and older populations [88,89]. Rupture is the main AAA complication, with only ~20% chance of survival [90]; however, cardiovascular events are the most common cause of death in these patients, with increased cardiovascular death risk by approximately 3% per year after AAA diagnosis [91].

Receiving far less attention than other CVDs, probably due to the difficulty of diagnosis, AAA lacks accurate and reliable detection, risk stratification, and rupture prediction tools [92], while emerging evidence supports the potential use of molecular biology to act as biomarkers or therapeutic approach in this condition [93].

Zhou et al., [94] screened the circRNA expression profile in aortas from four AAA patients. RNA high-throughput sequencing detected 13,295 circRNAs, with 411 differentially expressed (145 upregulated and 266 downregulated) compared to control. The relative downregulated expression of hsa_circ_0005360 in these aortic tissues, confirmed by qRT-PCR, reveals the potential role of this circRNA in AAA pathogenesis since it is an alternatively transcript of AAA-associated LDLR gene [95]. In addition, circRNA/miRNA interaction analysis predicted one binding site between hsa_circ_0005360 and miR-181b, with miRNA associated with vascular inflammation, atherosclerosis, and aneurysms [96,97]. This same expression profile study was added to an integrated analysis [98] compiling four gene expression datasets, including 23 normal artery tissues and 97 AAA samples, and using different prediction tools (e.g., Gene Ontology, Kyoto Encyclopedia of Genes and Genomes analysis, Targetscan, and CircInteractome). The majority of differently expressed genes (DEGs) (263 upregulated and 177 downregulated) were associated with extracellular matrix, tumor necrosis factor-α (TNF-α), and transforming growth factor-β (TGF-β). Bioinformatic analysis, used to build an interaction network between circRNAs, miRNAs, and mRNAs, revealed that the circ_0005073 may modulate several miRNAs affecting the expression of important DEGs in AAA, such as CCL2 (macrophage infiltration), SPP1 (proinflammatory), and UBA52 (protein degradation).

**Figure 2:**
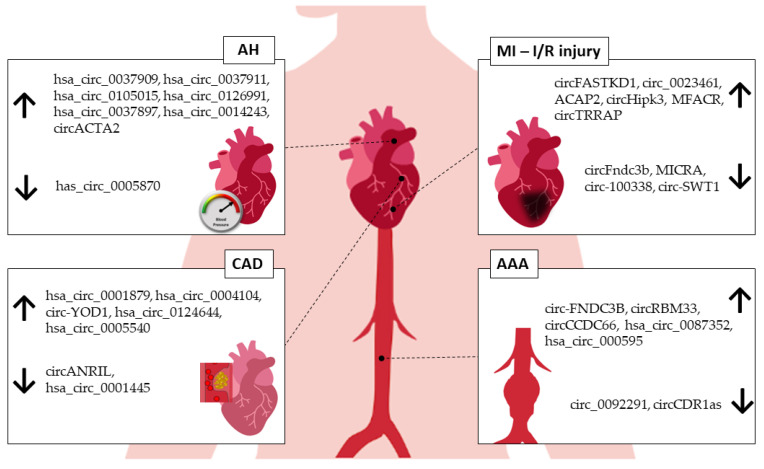
circRNAs associated with human CVDs. AH—arterial hypertension, MI—myocardial infarction, I/R—ischemia/reperfusion injury, CAD—coronary artery disease, AAA—abdominal aortic aneurysm. ↑—upregulated, ↓—downregulated.

AAA has a complex and multifactorial pathogenic process. Although the literature addressing circRNAs specifically in AAA is still scarce (Table 4) (Figure 2), the current evidence strongly supports the role of circRNAs in cellular processes that could be associated with the development of AAA, such as the modulation of endothelial cells, macrophages, and VSMCs [99].

## 7. Exercise-Related circRNAs in the Cardiovascular System

Previous studies reviewed the literature focusing on how CVS-related ncRNA expression responds to physical exercise. The conclusion is quite similar between them: although there is great interest in this topic, we lack studies aiming to describe these underlying molecular mechanisms. Aerobic exercise improved cardiac function and decreased cardiac apoptosis and fibrosis by modulating the expression of a few long noncoding RNAs (lncRNAs): growth arrest specific 5 (GAS5), myocardial-infarction-association transcript (MIAT), metastasis-associated lung adenocarcinoma transcript 1 (MALAT1), and H19 (encoded by the H19 gene) [111]. Targeting another class of ncRNAs, Wang et al., [112] described exercised-regulated miRNAs that benefit the heart by promoting protective effects in myocardial fibrosis (miR-29a and miR-29c), ischemia-reperfusion injury (miR-17-3p and miR-222), myocardial infarction (miR-1, miR-214), and heart failure (miR-21-5p, miR-132-3p, miR-208b-3p, miR-212-3p, and miR-335). The majority of studies focus on running and swimming exercise.

With regard to physical-exercise-related circular RNA expression, the evidence is even scarcer. Guo et al., [113] compared the skeletal muscle circRNA profile in sedentary young versus aged mice and aged mice under aerobic training and identified circBBS9 as a potential biomarker of aging-associated muscle dysfunction regulated by exercise. Niu et al., [114] found reduced expression of circRIMS2 and brain-derived neurotrophic factor (BDNF) in the serum of patients with vascular cognitive impairment (VCI) and experimentally showed that aerobic exercise improved cognitive function by inhibiting neuronal apoptosis through the circRIMS2/miR-186/BDNF axis. In another study, aerobic exercise combined with glucosamine therapy improved knee osteoarthritis in a rabbit model by enhancing the expression of CircUNK and, consequently, regulating cartilage-differentiation- and apoptosis-related genes. Meinecke et al., [115] explored the circRNA expression profile in the plasma of marathon runners and proposed circMBOAT2 as a promising biomarker for cardiopulmonary adaptation.

Recently, Zhu et al., [116] showed the role of the circRNA Circ-Ddx60 in heart failure using physical exercise as preventive treatment. The rationale of this study is based on the report in which physical exercise provided an antihypertrophic memory to myocardial muscle even after the regression of exercise-induced physiological hypertrophy [117]. In this study, the term exercise hypertrophic preconditioning (EHP) was applied, as preventive intervention, and was followed by a transverse aortic constriction (TAC) or sham surgery. The EHP group showed an increased expression of Circ-Ddx60, and its silencing attenuated the antihypertrophic effect of exercise, worsening heart failure in animals that underwent TAC. 

To the extent of our knowledge, this is the first research article to explore the potential of exercise-related circRNA in a cardiovascular disease, providing interesting insights in this vast research field.

## 8. Conclusions

Discovered in the 1970s, circRNAs were thought to be nothing more than splicing errors until the early 2010s. Recently, with proper technology and advances in molecular biology research, circRNAs have become a hot topic in health science. The study of circRNA function in physiological and pathological conditions seems to have great potential to improve the understanding of the underlying mechanisms in several aspects of the cardiovascular system, which could be applied in the diagnosis and treatment of cardiac-related diseases.

Some interesting characteristics, such as high stability and resistance, abundance, and sex-, age-, tissue-, and cell-specific expression, make circRNAs a promising tool for diagnostic and prognostic biomarkers. On the other hand, their involvement with some crucial cell processes, such as proliferation, migration, differentiation, apoptosis, and autophagy, suggests that circRNAs play important roles in the development and progression of diseases.

Ultimately, the current literature exploring the role of physical exercise/training as a nonpharmacological intervention, extensively evidenced in other health/performance scenarios, is still scarce when related to circRNAs.

## 9. Perspectives

Despite these advances, it is still difficult to translate circRNA findings into clinical practice. The lack of standardized procedures, small-sample-size cohort studies, and other minor issues challenge their use in current medicine.

As quoted before, circRNAs opened “a new era in the study of CVDs” [118], and we are just beginning explore and understand this vast field.

## Figures and Tables

**Table 1:** List of circRNAs associated with arterial hypertension.

Id	Host Gene	Species	Source	Expression	Action	Ref.
hsa_circ_0037909	GSPT1	Human	Blood, HAECs, HUVECs	Up	Sponges hsa-miR-637, possibly impacting in LDL and serum creatinine concentrations.	[29]
hsa_circ_0037911	GSPT1	Human	Blood	Up	Biomarker.	[30]
rno_circ_006016	Erc2	Rat	Kidney	Down	Controls blood pressure by multiple circRNA–miRNA–gene interactions.	[31]
hsa_circ_0105015	GSPT1	Human	Blood Endothelial cells	Up	Promotes target hsa-miR-637 to activate the inflammatory pathway.	[32]
hsa_circ_0126991	SEPT11	Human	Blood	Up	Biomarker.	[33]
rno_circ_0009197	Cdh23	Rat	Aortic vascular tissues	Down	Aortic circRNAs play potential roles in regulating hypertensive vascular remodeling and dysfunction.	[24]
rno_circ_0005818	Dnajc1			Up		
rno_circ_0005304	-			Up		
rno_circ_0005506	Ryr2			Up		
rno_circ_0009301	-			Up		
circACTA2	ACTA2	Human	Artery tissues	Up	Promotes vascular smooth muscle cell senescence by targeting circACTA2/ILF3/CDK4 axis.	[25]
		Human	HASMCs	Up	Regulates α-SMA expression.	[28]
hsa_circ_0037897	GSPT1	Human	Blood	Up	May be involved in hypertension by sponging hsa-miR-145-5p.	[34]
hsa_circ_0014243	CHTOP	Human	Blood	Up	Plays a crucial role in genesis and development of hypertension. Could be used as biomarker.	[35]
has_circ_0005870	SETD2	Human	Plasma	Down	Biomarker.	[36]

HAECs—human aortic endothelial cells; HUVECs—human umbilical vein endothelial cells; HASMCs—human aortic smooth muscle cells.

**Table 4:** List of circRNAs associated with AAA.

Id	Host Gene	Species	Source	Expression	Action	Ref.
circChordc1	Chordc1	Mouse	Abdominal aorta	Down	Improves VSMCs growth, suppressing aneurysm formation and reducing the risk of rupture by inducing vimentin degradation.	[100]
circ-FNDC3B	FNDC3B	Human	Aortic tissue	Up	Promotes VSMCs inflammation and oxidative stress through miR-143-3p/ADAM10 axis, leading to the development of AAA.	[101]
circRasGEF1B	Rasgef1b	Mouse	Abdominal aorta	Up	circRasGEF1B-ZFP36 axis mediates macrophage-induced VSMC apoptosis in an Sm22α^-/-^ mice AAA model.	[102]
circ_0092291	EIF2S2	Human	Blood HAVSMC	Down	Inhibits AAA-associated cell damage via circ_0092291/miR-626/COL4A1 axis.	[103]
circCdyl	Cdyl	Mouse	Abdominal aorta	Up	Promotes vascular inflammation through M1-type macrophages polarization, inducing AAA formation.	[104]
circRBM33	RBM33	Human	Abdominal aorta	Up	Promotes ECM degradation in aorta-isolated VSMCs via the miR-4268/EPHB2 axis.	[105]
circCCDC66	CCDC6 6	Human	VSMCs	Up	Upregulates its host gene, modulating VSMCs proliferation and apoptosis and facilitating AAA development.	[106]
circCBFB	CBFB	Human	HAVSMC	-	Its suppression increased the expression of miR-28-5p, promoting apoptosis of VSMCs.	[107]
hsa_circ_0087352	UBQLN1	Human	THP-1 cells HAVSMC	Up	Promotes inflammatory response in macrophages, enhancing the expression and secretion of IL-6 and TNF-α by sponging hsa-miR-149-5p.	[108]
CircCDR1as	CDR1	Human	Aortic tissues VSMCs	Down	Regulates VSMC apoptosis and proliferation via circCDR1as/miR-7/CKAP4 axis.	[109]
hsa_circ_000595	-	Human	Aortic tissues HAVSMC	Up	Its knockdown decreased apoptosis in HAVSMC.	[110]

VSMC—vascular smooth muscle cell; AAA—abdominal aorta aneurysm; Sm22α^-/-^—smooth muscle knockout; HAVSMC—human aortic vascular muscle cell; ECM—extracellular matrix; THP-1—human leukemia monocytic cell line; IL-6—interleukin 6; TNF-α—tumor necrosis factor alpha.
